# Sensitivity of physiotherapy-based clinical tests in detecting change in gait and balance performance following a 50 mL CSF tap test in idiopathic normal pressure hydrocephalus

**DOI:** 10.1186/s12987-026-00776-8

**Published:** 2026-02-17

**Authors:** Kardelen Akar, Lena Kollén, Mats Tullberg, Hanna C. Persson

**Affiliations:** 1 Hydrocephalus Research Unit, Department of Clinical Neuroscience, Institute of Neuroscience and Physiology, Sahlgrenska Academy, University of Gothenburg, Gothenburg, Sweden; 2 Graduate School of Health Sciences, Koç University, Istanbul, Türkiye; 3 Department of Occupational Therapy and Physiotherapy, Sahlgrenska University Hospital, Gothenburg, Sweden; 4 Department of Neurology, Sahlgrenska University Hospital, Gothenburg, Sweden; 5 Research Group of Rehabilitation Medicine, Sahlgrenska University Hospital, Gothenburg, Sweden

**Keywords:** Idiopathic normal pressure hydrocephalus, Cerebrospinal fluid, Lumbar puncture, Tap test, Postural function, Balance, Walk test, Physical therapy modalities, Outcome assessment, Treatment effectiveness

## Abstract

**Background:**

Cerebrospinal fluid tap test (CSF TT) is a key predictive method commonly used to identify candidates for shunt surgery in idiopathic Normal Pressure Hydrocephalus (iNPH), however, the sensitivity of this procedure is limited. The aim was to compare sensitivity of a broad set of physiotherapy-based clinical tests for assessing changes following CSF TT in a group of selected iNPH patients and to explore potential sex-based differences in the responses.

**Methods:**

Ninety-five selected iNPH patients (mean age 77, SD 6, 57% male), underwent CSF TT prior to shunt surgery. Clinical tests comprised different static balance tests in varying conditions including feet and heels apart and together; eyes open and closed; on floor and foam cushion, followed by gait and functional performance measures including 10-meter walk test (10MWT), 3-meter backward walking (3MBW), Timed-up and Go (TUG) and 6-minute walking test (6MWT). Duration in seconds (sec) and distance were noted. Assessments were conducted at set time points before and after the drainage procedure. Changes in performance were analyzed, and responses compared between sexes.

**Results:**

Significant increase was observed across most static balance tests except tandem eyes closed (EC) and one-legged stance tests. The largest enhancements were seen in the Romberg test (4.9 s), followed by foam cushion-eyes open (EO) tests (feet together: 3.9 s and heels together: 3.8 s), heels together-EC (3.8 s) and tandem-EO (3.1 s). Gait and functional tests significantly improved (*p* < 0.001) and 3MBW showed the largest change, with a 32% reduction in sec and a 23% reduction in steps, followed by decrease in TUG sec (27%) and 10-MWT sec (21%), and 6MWT distance increase (25%). Males demonstrated significant increase in heels together-EO and feet together-EO duration which were not observed in females; after adjusting for baseline performances, no significant sex-related differences in responsiveness to CSF TT were found.

**Conclusions:**

Challenging comprehensive physiotherapy tests, including gait assessments in direction and capacity, such as 3MBW and 6MWT, together with postural control evaluations using foam cushion and tandem stance increases the sensitivity to change and are suggested to be used to detect functional changes after CSF TT in patients with iNPH.

**Trail registration:**

The project is registered in "FoU inSweden” (Research and Development in Sweden) ID: 285356.

**Supplementary Information:**

The online version contains supplementary material available at 10.1186/s12987-026-00776-8.

## Background

Idiopathic Normal Pressure Hydrocephalus (iNPH) is a common neurological condition among people aged 65 or above [1] characterized by ventriculomegaly presenting with gait and balance disturbance, cognitive impairment and urinary incontinence [2]. Gait is typically slow, broad-based and shuffling, often accompanied by freezing of gait and paratonia of the limbs [3, 4] with increased double support phase and a decreased toe-off angle, yielding a typical magnetic gait pattern [5]. Static and dynamic balance deficits, including retropulsion, are hallmark symptoms often serving as a primary indicator of gait problems [6]. As the disease progresses, gait variability and dynamic balance impairments worsen, significantly increasing instability and the risk of falls, particularly in older adults [7].

Diagnosing iNPH involves a comprehensive clinical evaluation, including a neurological examination to assess symptoms, and magnetic resonance imaging (MRI) and/or computed tomography (CT) to visualize anatomy of the ventricular enlargement and potential obstruction [2, 8]. The cerebrospinal fluid tap test (CSF TT), involving the removal 30–50 ml of CSF via lumbar puncture (LP) [9, 10], serves as an important diagnostic supplementary method, particularly recommended for patients exhibiting the cardinal symptoms alongside atypical presentations or coexisting neurological conditions [11]. A detailed physiotherapy assessment encompassing measures of gait, balance and functional performance can be employed to evaluate changes following CSF TT. While the 10-meter walk test (10 MWT) [12–18] and sometimes the Timed-Up and Go (TUG) test [18–20] are commonly used, these two tests may not fully capture the spectrum of functional changes. In the post-CSF TT, improvements in gait speed are often observed initially [21], followed by increased stride length, reduced double support and stance phases, increased swing phase, and more regular strides indicative of a positive response and potential candidacy for shunt surgery [22]. The lack of inconsistencies in defining improvement thresholds for gait speed and duration [9, 22–24], as well as in the standardized timing of post-assessment [25], presents challenges in accurately identifying CSF TT responders. Additionally, false-negative CSF TT results can lead to exclusion of patients who could benefit from shunt surgery [11]. While CSF TT can be a valuable adjunct, especially in patients with advanced symptoms, clinical judgement remains crucial, and the decision to use CSF TT should be tailored to the individual. In our iNPH center, given the reported low sensitivity of the CSF-TT [11, 17, 23], the test is not used routinely but rather selectively, guided by clinical presentation and including both gait and balance performance to inform shunt surgery candidacy.

This study aimed to compare the sensitivity of a broad set of physiotherapy-based clinical measures for detection of changes following CSF TT in a group of selected iNPH patients, as well as to explore potential sex-based differences in the responses.

## Methods

### Patients

This hospital-based, retrospective cohort study was conducted at Sahlgrenska University Hospital, Gothenburg, Sweden, with consecutive inclusion of participants diagnosed with iNPH according to international guidelines [2] from January 2017 until December 2023 and subjected to shunt surgery before September 2024.

All included patients underwent a standardized CSF TT as part of the diagnostic work-up, mainly due to indistinct or atypical symptomatology, before shunt surgery. Patients subjected to shunt surgery without a prior CSF TT (criteria specified below), as well as those unable to perform at least one of the standardized physiotherapy assessments during the pre and/or post-CSF TT, were excluded from the study.

### Diagnostic procedure

Patients with suspected iNPH underwent a standardized multiprofessional team-based evaluation at the iNPH center. This evaluation involved comprehensive assessments, including examinations by a neurologist, a physiotherapist, a neuropsychologist, and a specialist nurse, and had an MRI of the brain and blood and lumbar puncture with measurement of the CSF opening pressure and laboratory tests. The assessments were followed by a multidisciplinary team discussion represented by a neurologist, a nurse, a physiotherapist, a neuropsychologist and a neurosurgeon to review the findings and make a conclusion regarding diagnosis and treatment. Based on the team discussion, patients were either directly referred to shunt surgery, when diagnosis was deemed certain and outcome of shunt surgery favorable based on clinical symptoms, MRI findings or laboratory test results, or underwent additional testing with the CSF TT as a supplementary test when the outcome of shunt surgery was deemed uncertain due to inconsistent clinical symptoms, MRI findings, or laboratory test results: patients showing a positive CSF TT being referred to shunt surgery. This workup, adding additional predictive tests as a second intervention, is based on earlier reports [10, 26] and our experience of operating on patients based on typical clinical and MRI features. This study exclusively included patients who underwent CSF TT and subsequently received a shunt (Strata valve, Medtronic, Goleta, USA; programmed at setting 1.5).

### CSF TT procedure

The CSF TT procedure was modified from the protocol by Wikkelsö et al. [9]. On day 1, the pre-tap physiotherapy assessment was conducted at 2:00 PM, comprising standardized tests described below. On day 2 at 9:00 AM, an LP was performed with the patients in the lateral recumbent position, followed by measurement of opening pressure and withdrawal of 50 mL of cerebrospinal fluid [9]. The post-tap physiotherapy assessment was conducted on day 2 at 2:00 PM, using the same standardized protocol. The CSF TT was deemed positive based on a holistic evaluation conducted during multiprofessional team conferences, typically when significant improvements were observed in more than one test, as supported by video assessments of changes in gait quality and performance.

### Pre-and post-CSF TT physiotherapy assessments

The assessment was performed in a separate calm room according to a standardized procedure and included assessment of static balance, gait and functional measures. The physiotherapist stood by or walked with the patient during all tests to prevent falls. The patient’s best performance in each test was strived for.

#### Static balance measurements

The static balance assessment consisted of stance tasks performed without shoes as follows: arms by side (1) heels together-eyes open (EO); (2) heels together-eyes closed (EC); (3) feet together-EO; (4) Romberg’s test [27]; (5) heels together on a foam cushion-EO; (6) heels together on foam cushion-EC; (7) feet together on foam cushion- EO; (8) Romberg’s test on foam cushion; (9) tandem stance with self-selected foot front-EO, (10) tandem stance-EC, and 11) right leg stance-EO; 12) left leg stance-EO. In all static balance tests, the duration of maintained stance in seconds until the patients either opened their eyes (in tests with eyes closed), needed support or lost balance with a maximum of 30 s (sec) were recorded.

#### Gait measurements

All short gait measures were performed twice. Duration was measured in sec, and the number of steps was counted. The use of walking aids, if needed, was accepted.

The 10MWT was assessed at self-selected speed (10MWT-SS) and at maximum speed (10MWT-MS): Tests were performed from a stationary position at a 10-meter distance between two markings on the floor. Test instructions for the 10MWT-SS was to walk at a comfortable pace and for the 10MWT-MS to walk as quickly, still safely, as possible [28]. Running was not allowed. The best of two trials was noted for 10MWT-SS and the mean result of two was used for 10MWT-MS.

For the 3-meter backward walking (3MBW) the patient was instructed to walk backwards quickly but with a safe pace for a 3-meter distance starting from a stationary position [29]. The mean time (sec) of two trials and corresponding number of steps were registered.

The 6-minute walking test (6-MWT) was performed once, along a 30-meter corridor marked by two cones. Patients were instructed to complete as many laps as possible within six minutes. The total distance and duration were recorded in meters. Patients were allowed to pause in an upright position (including leaning towards the wall) if needed. If the patient needed to sit down and rest, the test was ended [30].

#### Functional measurements

The TUG was performed with the patient rising from a standard-height (45 cm) chair, permitted to use armrests for support. Patients walked 3 m at a self-selected speed, passed and turned at a marker on the floor, walked back and sat down on the chair. The time was registered in sec from when the patient rose from the chair and until again touching the chair [31]. The number of steps was counted and mean of two trials was recorded.

The 30-second chair stand test (30sCST) was evaluated for functional lower-limb strength. Patients were instructed to cross their arms on their shoulders, then stand up straight and sit down as many times as possible during 30 s on a standard chair with arm support. The number of full stands were noted [32].

### Other measures

Baseline demographic data (age, sex, body mass index (BMI), disease duration and comorbidities), fall accidents during the last three months before the initial visit, and type of shunt surgery operation were registered. Other baseline clinical measurements included the Gothenburg iNPH scale introduced by Hellström, total score and domain sub-scores (gait, neuropsychology, balance and continence). The Gothenburg iNPH-scale yields a score of 0-100 where 100 represents the performance of age-matched healthy individuals. Overall postoperative improvement was defined as a postoperative increase in total iNPH scale score of at least 5 points [33]. Further, screening of cognitive function with Mini-Mental State Examination (MMSE) score [34], global functional assessment based on the modified Rankin Scale (mRS) [35], and the level of physical activity according to Saltin Grimby Physical Activity Scale (SGPALS) [36] were assessed.

Postoperative follow-up assessment was conducted using the Gothenburg iNPH scale, and improvement was defined as an increase of five points or more in the total score compared to the baseline assessment.

### Statistical analysis

Statistical analysis was performed using IBM SPSS Statistics for PC version 29. Descriptive statistics were presented using the mean and standard deviation (SD) for normally distributed data or median and interquartile range (IQR) for non-normally distributed data. Categorical variables are presented in numbers and percentages. Between groups differences were assessed using Student t-test, Mann Whitney U-test (MWU), chi-square test, or Fisher’s exact test as appropriate for the variable type and distribution. Normality was assessed using the Kolmogorov-Smirnov test.

Changes in static balance, gait and functional test performance following CSF TT were analyzed using Welch’s t-test. Linear mixed models were applied to account for repeated measures and handle missing data, with time (pre vs. post) as the independent variable. No additional covariates were included in the model. Pre-and post CSF TT assessments were presented as mean and standard deviation for static balance measures, and as geometric mean and coefficient of variation (CV) for gait and functional measures. Change after CSF TT was expressed as mean difference for static balance measures, and as fold change (after log transforming) for gait and functional measures.

Sex-based differences in physiotherapy measures were analyzed using adjusted mean differences (95% of Confidence Interval) (CI) for changes in static balance measures and adjusted fold changes (95% CI) for gait and functional measures. These comparisons were performed using Student’s t-test. Tests were two-sided and *p* < 0.05 was considered statistically significant.

## Results

### Patient characteristics

**Fig. 1:**
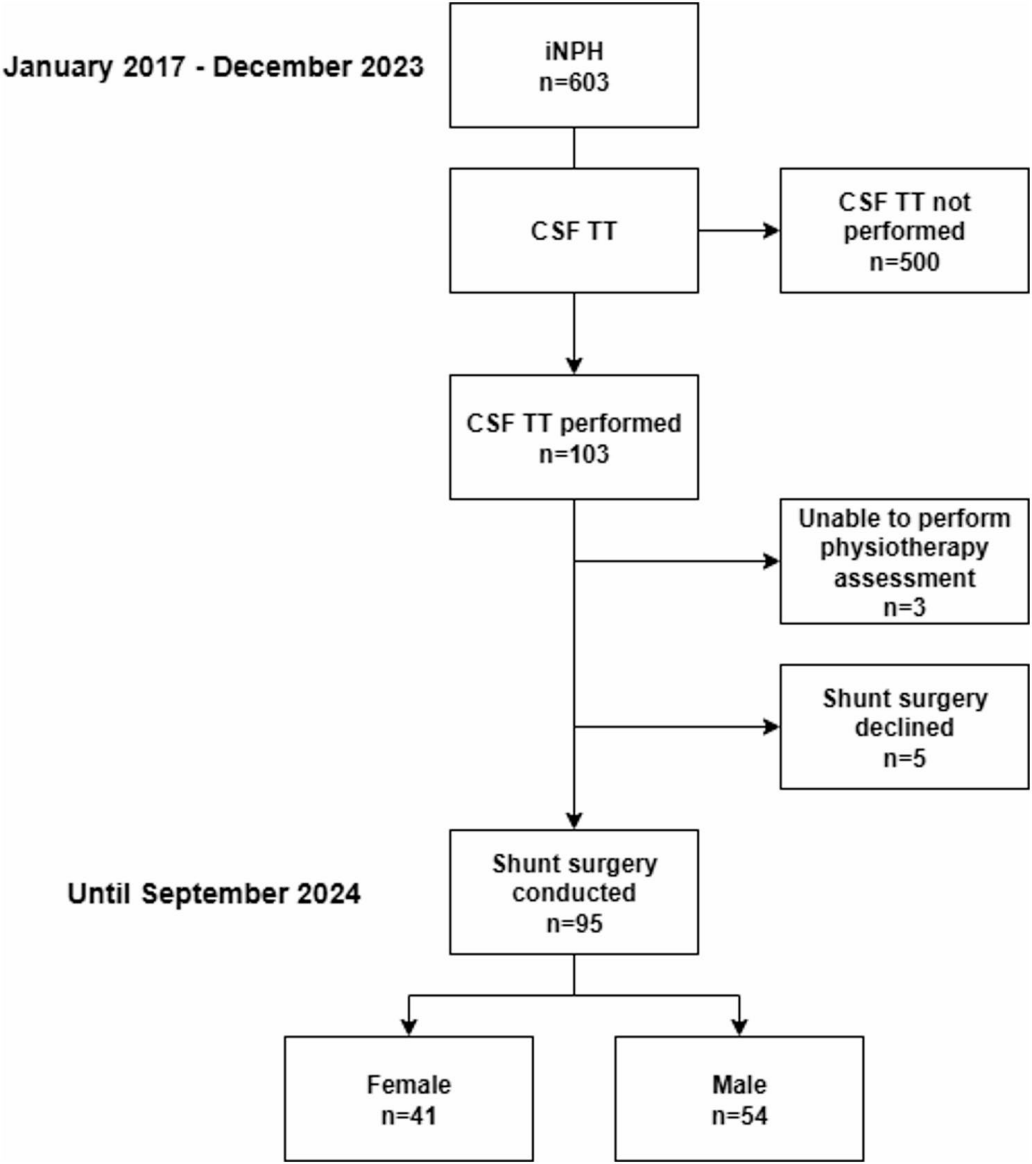
A flowchart depicts the selection process of patients included in the study. CSF TT, Cerebrospinal fluid tap test

In total, 603 consecutive patients were diagnosed with iNPH. Of these, 103 patients underwent the CSF TT procedure before surgery. Ninety-five patients participated in both pre- and post CSF tap physiotherapy assessments and did not undergo additional invasive diagnostic test for and subsequently proceeded to shunt surgery and were included in the present study (Fig. 1). Overall postoperative improvement on the iNPH scale at median 6 (IQR 4–10) months follow-up (*n* = 84) was seen in 54.8% of patients: preoperative score (mean ± SD) 50.2 ± 16.1; postoperative score 56.1 ± 22.4, *p* = 0.002).

At the time of pre assessment the participants mean (SD) age was 76 (6) years, and 41 (43%) were females. The mean duration of symptoms was 36 months. The majority, 90 patients received a ventriculoperitoneal (VP) and 5 patients a ventriculo-atrial (VA) shunt. No differences in baseline characteristics between sexes were present except for vascular risk factors which were higher in males, and balance impairment which was more abundant/pronounced in females (Table 1).

**Table 1:** Characteristics of study Participants, stratified by sex

		Total *n* = 95	Female *n* = 41	Male *n* = 54	*p*-value
Age, years (Mean, SD)		77(6)	78 (6)	76 (5)	ns
Sex, male (M%)		54 (57)	-	-	-
BMI, kg/m^2^ (Median, IQR)		26 (24–28)	25 (21–28)	26 (24–28)	ns
Disease duration, months (Median, IQR)		36 (24–60)	42 (24–60)	36 (24–60)	ns
Comorbidities/ disease, n (%)	Hypertension	63 (66)	25 (61)	38 (70)	ns^a^
	Diabetes	24 (25)	11 (27)	13 (24)	ns^a^
	Cardiovascular	29 (31)	8 (20)	21 (39)	**0.04** ^a^
	Hyperlipidemia	34 (45)	13 (42)	21 (48)	ns^a^
	Ischemic heart dis.	9 (12)	2 (7)	7 (16)	ns^b^
	Other heart dis.	18 (23)	4 (13)	14 (29)	ns^b^
	Cerebrovascular dis.	22 (28)	9 (28)	13 (27)	ns^a^
	Any vascular risk	58 (77)	20 (65)	38 (84)	**0.04** ^**a**^
	Other neurological	21 (34)	11 (41)	10 (29)	ns^a^
Number of falls within 3 months (Median, IQR)		0 (0–2)	1 (0–1)	0 (0–3)	ns
Shunt surgery type, n (%)	VP	90 (95) 5 (5)	37 (90) 4 (10)	53 (98) 1 (2)	ns^b^
	VA				
iNPH scale, score (Mean, SD)	Total	50 (16)	47 (17)	53 (15)	ns
	Gait domain	44 (23)	41 (23)	47 (22)	ns
	Neuropsychologic domain	46 (21)	46 (22)	47 (21)	ns
	Balance domain	61 (20)	55 (24)	65 (16)	**0.02**
	Continence domain	55 (26)	52 (24)	57 (28)	ns
MMSE score (Median, IQR)		24 (21–28)	24 (21–27)	26 (21–28)	ns
mRS (Median, IQR)		3 (2–4)	3 (2.5-4)	3 (2–3)	ns
SGPALS (Median, IQR)		1 (1–2)	1 (1–2)	1 (1–2)	ns

Data are presented with mean (SD) or median (interquartile 25th-75th range). P-values given for comparisons between males and females

Abbreviations: ns, not significant; BMI, Body Mass Index; VP, Ventriculoperitoneal; VA, Ventriculoatrial; dis, disorder; Other neurological, Other comorbid neurological disorder; iNPH, idiopathic normal pressure hydrocephalus; MMSE, Mini-Mental State Examination; mRS, modified Rankin Scale; SGPLAS, Saltin-Grimby Physical Activity Level Scale

^a^, chi-square test; ^b^, Fisher’s exact test

### Changes in balance and gait test performance after CSF TT

Significant improvements in duration between pre- and post CSF TT performance were seen in all balance tests except for tandem-EC and standing on one-leg after CSF TT (Table 2).

Repetition of stands in 30-sCST increased with a mean of 1.1 times (*p* < 0.001).

**Table 2:** Pre- and Post-CSF TT static balance tests

	Number of missing values	Pre-CSF TT	Post-CSF TT	Mean difference (95% CI)	*p*-value
Heels together-EO, sec	3	26 (9)	28 (7)	2.5 (0.9–4.1)	**0.003**
Heels together-EC, sec	5	19 (12)	23 (11)	3.8 (1.9–5.8)	**< 0.001**
Feet together-EO, sec	3	24 (11)	26 (9)	2 (0.4–3.6)	**0.016**
Romberg, sec	6	14 (13)	19 (12)	4.9 (3.1–6.7)	**< 0.001**
Heels together-foam cushion-EO, sec	11	17 (14)	21 (13)	3.8 (1.9–5.8)	**< 0.001**
Heels together-foam cushion-EC, sec	18	6 (10)	9 (11)	2.7 (1.2–4.2)	**< 0.001**
Feet together-foam cushion-EO, sec	13	14 (14)	19 (14)	3.9 (1.8–6)	**< 0.001**
Romberg-foam cushion, sec	17	3 (7)	6 (10)	2.9 (1.5–4.3)	**< 0.001**
Tandem stance-EO, sec	15	5 (8)	8 (11)	3.1 (1.6–4.6)	**< 0.001**
Tandem stance-EC, sec	16	1 (2)	1 (2)	0.3 (-0.1–0.7)	ns
Right leg stance-EO, sec	16	2 (5)	2 (6)	0.5 (-0.2–1.1)	ns
Left leg stance-EO, sec	15	1 (4)	2 (4)	0.4 (-0.6–1.3)	ns

Values were presented with mean (SD)

Abbreviations: EO, Eyes open; EC, Eyes closed; sec, seconds

After CSF TT, significant improvements were observed in all gait and functional tests (*p* < 0.001) (Table 3). The proportion of participants exhibiting improvement ranged from 77% for 10MWT-SS (steps) to 93% for TUG (sec).

The 3MBW showed the largest fold change with 32% reduced duration (sec) and 23% reduced steps. This was followed by reduced TUG (sec) with 27% and 10-MWT-SS (sec) with 22% (Table 3).

**Table 3:** Pre- and Post-CSF TT gait and functional test results in the total patient group

	Number of missing values	Pre- CSF TT	Post- CSF TT	Fold change (95% CI)	*p*-value	Improved, *n* (%)	*p*-value
10MWT-SS duration, sec	5	17 (0.5)	13 (0.4)	0.8 (0.7–0.8)	**< 0.001**	77 (86%)	**< 0.001**
10MWT-SS steps	5	25 (0.4)	22 (0.3)	0.9 (0.8–0.9)	**< 0.001**	69 (77%)	**< 0.001**
10MWT-MS duration, sec	23	11 (0.4)	9 (0.3)	0.9 (0.8–0.9)	**< 0.001**	56 (78%)	**< 0.001**
10MWT-MS steps	22	20 (0.3)	18 (0.2)	0.9 (0.9–0.9)	**< 0.001**	58 (79%)	**< 0.001**
3MBW duration, sec	7	16 (0.8)	11 (0.6)	0.7 (0.6–0.7)	**< 0.001**	76 (86%)	**< 0.001**
3MBW, steps	7	21 (0.6)	16 (0.5)	0.8 (0.7–0.8)	**< 0.001**	75 (85%)	**< 0.001**
TUG duration, sec	3	26 (0.8)	19 (0.6)	0.7 (0.7–0.8)	**< 0.001**	86 (93%)	**< 0.001**
TUG, steps	4	26 (0.5)	21 (0.4)	0.8 (0.8–0.9)	**< 0.001**	73 (80%)	**< 0.001**
6 MWT distance, m	10	179 (0.8)	223 (0.8)	1.3 (1.2–1.3)	**< 0.001**	76 (89%)	**< 0.001**

Descriptive data are presented as geometric mean (CV)

Abbreviations: 10MWT-SS, 10-meter walk test at self-selected speed; 10MWT-MS, 10-meter walk test at maximum-speed; 3MBW, 3-meter backward walking; TUG, Timed-up and go; 6MWT, 6-meter walking test; CI, confidence interval; FC, fold change; sec, seconds; m, meter

### Sex differences at baseline and following CSF TT

**Fig. 2:**
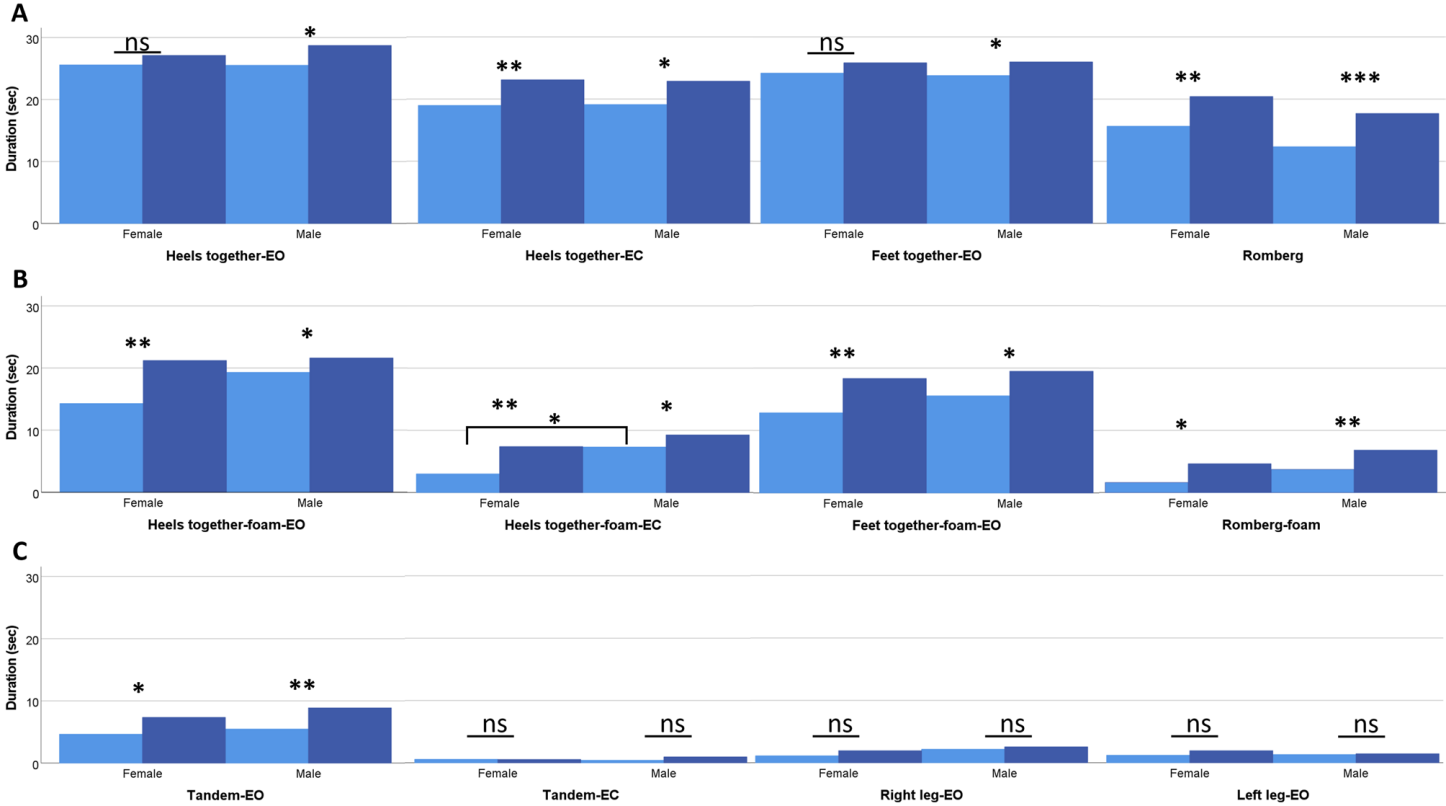
Changes in Static Balance Test Performance on Ground (**A**), on Foam Cushion (**B**), and Tandem and One-leg tests (**C**) Following Cerebrospinal Fluid Tap Test (CSF TT) in Female and Male Groups. Bars represent mean values. Data are presented for pre-CSF TT (lighter colors) and post-CSF TT (darker colors) assessments. *, *p* < 0.05; **, *p* ≤ 0.01; ***, *p* ≤ 0.001; ns, not significant

**Fig. 3:**
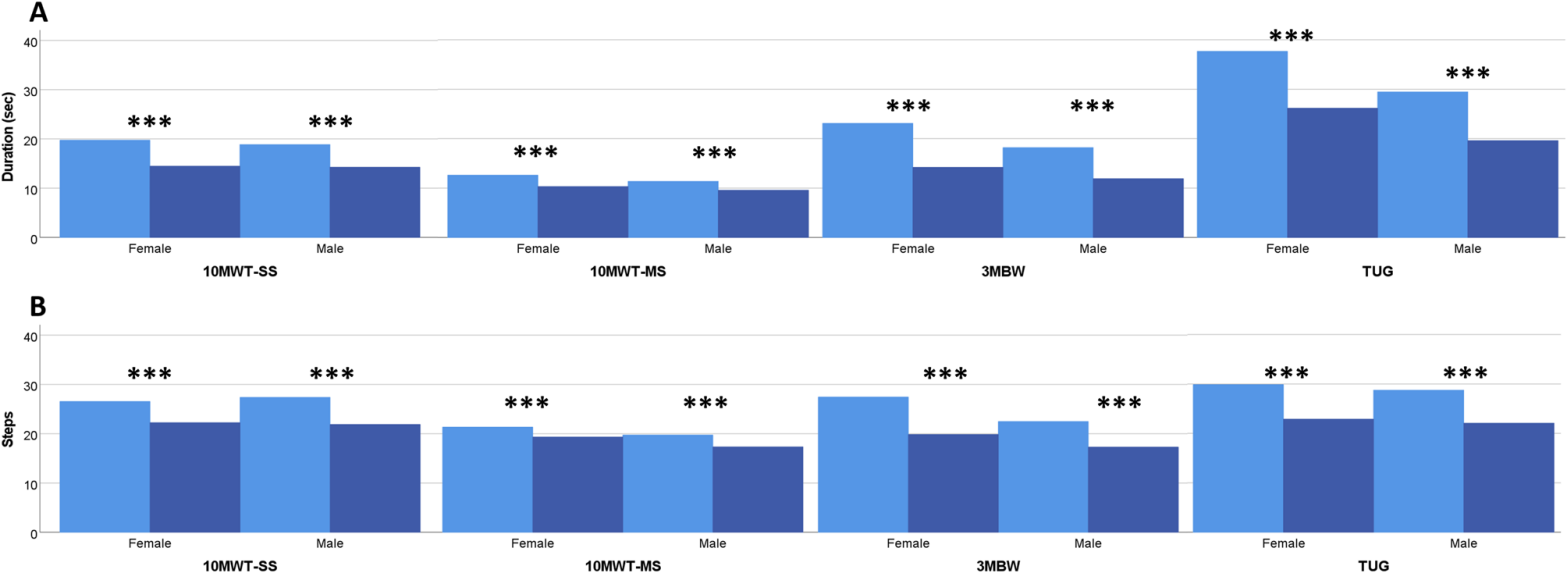
Changes in gait tests and timed up and go performance duration in sec (**A**) and steps (**B**) After cerebrospinal fluid tap test (CSF TT) in female and male groups. Bars represent mean values. Darker colors indicate post-CSF TT assessments. ***, *p* ≤ 0.001

**Fig. 4:**
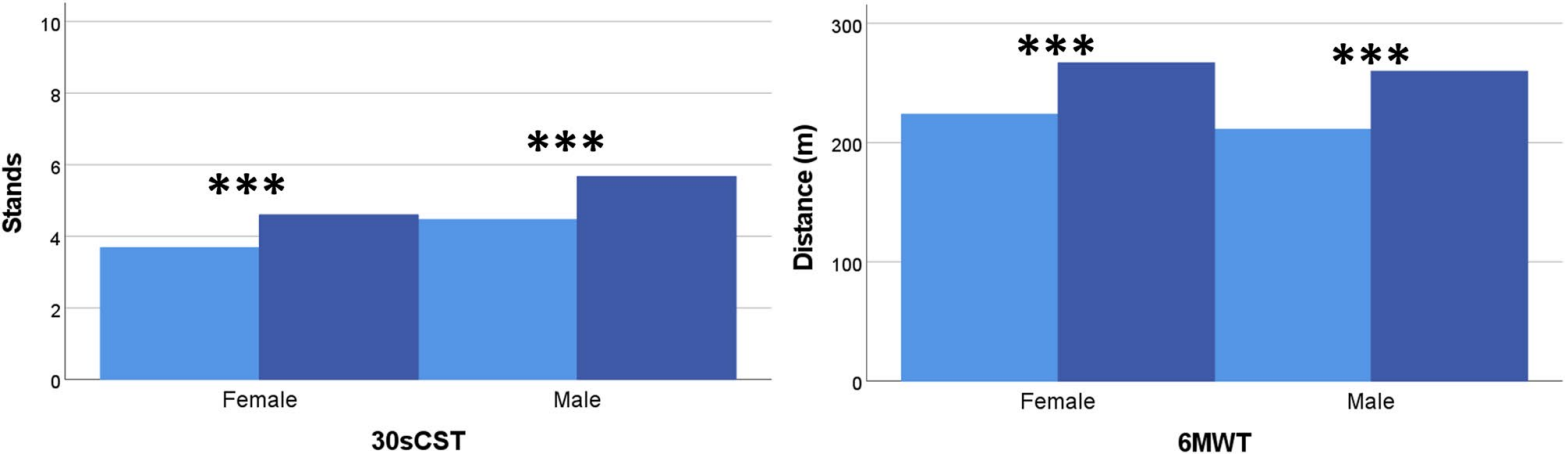
Changes in 30-second chair stand test (Left) and 6-minute walking test (Right) performance after cerebrospinal fluid tap test (CSF TT) in female and male groups. Bars represent mean values. ***, *p* ≤ 0.001

At baseline, there was no difference observed between sexes in the physiotherapy assessment except for females who performed significantly worse than males in heels together on foam cushion-EC test (Fig. 2). Significant improvements after CSF TT were seen in all tests, except for the tandem-EC and one-leg tests in both sexes and heels together-EO and feet together-EO tests in females. However, after adjusting for the pre-CSF TT performance no significant differences between sexes remained in any of the static balance, gait and functional tests (Figs. 2, 3, and 4).

After the CSF TT, the largest improvements were observed in the 3MBW test (sec) (reduction by 37% in females; 27% in males), followed by TUG (sec) (reduction of 27% in females; 26% in males) and 3MBW (steps) (reduction of 27% in females; 20% in males), increased 6 MWT distance (22% in females; 26% in males), and 10MWT-SS (sec) reduction of 23% in females and 20% in males.

## Discussion

This study investigated the responsiveness of a broad set of physiotherapy-based measurements following CSF tap test (CSF TT) in a selected group of patients with idiopathic normal pressure hydrocephalus (iNPH) and we explored potential sex-based differences. Improvements were observed in all outcome measures post- CSF TT, with the largest fold changes found in the 3-meter backwards walking (3MBW), the 6-minute walking test (6MWT) and in Timed Up and Go (TUG), outperforming the commonly used 10-meter walk test (10MWT).

In the majority of balance tests, improvements (increased duration) were observed after the CSF TT but were not significant for the tandem-eyes closed and one-leg stance conditions. The Romberg test yielded the largest absolute increase, followed by foam cushion tests (heels together and feet together) with eyes open, heels together eyes closed, and tandem stance-eyes open tests (Table 2).

While the Romberg test is often used in clinical settings, it exhibits limitation to detect subtle balance impairments [37]. In our cohort, the proportion of patients able to complete the 30-second Romberg test increased from 33% before CSF TT to 47% afterward. Similarly, Agerskov et al. [4] reported a significant post-shunt improvement in Romberg performance in iNPH patients-from 47% to 66%- noting that this increase was related to their impaired gait scores [4]. Conversely, standing on one-leg proves too challenging for many patients, also in our cohort performance where the mean duration was below three seconds. Our results suggest that standing on foam cushion tasks and tandem stance provides an effective alternative where Romberg and one-leg stance may be insufficient for detecting balance responsiveness after CSF TT.

Pre-CSF TT assessment showed that females had significantly lower duration in heels together on foam cushion-eyes closed test and did not demonstrate significant increase post-CSF assessment in heels together-eyes open and feet together-eyes open tests (Fig. 2). However, after adjusting for baseline performance, no significant sex-based differences were observed in the degree of change (Supplementary Table 1). This suggests that while baseline functional status may differ between sexes, sex is not a confounder in static balance outcome post-CSF TT. These results are consistent with a prior study suggesting that the iNPH balance domain preoperative performance is not predictive of surgical outcomes [38].

Improvements were observed across all gait tests after the CSF TT. Among them, the 3MBW and 6MWT demonstrated the highest fold change and thus responsiveness to CSF tap (Table 3). Interestingly, the self-selected speed 10MWT (10MWT-SS) demonstrated more substantial changes in duration compared to the maximum speed (10MWT-MS), contrasting with some studies suggesting 10MWT at maximum speed as a primary outcome for assessing surgical improvement [39, 40]. Our findings align more closely with Matsuoka et al. [41] who suggested the importance of test selection when interpreting CSF TT performance either for diagnostics or degree of disability with greater sensitivity to change in self-selected speed compared to 10MWT-MS.

The 3MBW was found to be especially relevant in iNPH population due to its unique capacity to predict fall risk in older adults [42], which may not be evident in shorter assessments. The test, which requires sustained walking, places greater reliance on proprioceptive input rather than visual feedback [43], thereby challenging the sensory integration process that is frequently altered in iNPH patients [44, 45]. Notably, backward walking demands higher-level motor planning and balance control and may reveal deficit not detected during forward gait alone [46]. Additionally, the inclusion of 3MBW in the protocol offers valuable insight into dynamic balance control and postural adaptability, both which are increasingly utilized in gait training and rehabilitation programs [47, 48].

Furthermore, the average increase of 44 m in the 6MWT (approximately 1.5 laps and 25% improvement) following CFS TT reinforces the utility of functional capacity assessments in this population. 6MWT captures endurance and walking efficiency, which are more reflective of real-life ambulation [49] and subtle gait disturbances in daily activities [50], especially with minor gait disturbances. Together the findings from 3MBW and 6MWT support the value of integrating both tests into routine evaluation protocol for iNPH, as they provide more sensitive complementary insights to substantial change following CSF TT.

A notable feature of this study is the standardized 4 h post-CSF tap assessment at the same time of day, 24 h after the baseline assessment. Our procedure is based on Wikkelsø et al. [9] and clinical experience. This interval aligns to conclusions drawn by Virhammar et al. [51] comparing response 2–24 h post TT, concluding that the CSF TT can be evaluated at any time within the first 24 h, which is also recommended in the Japanese Guidelines 3rd edition [10].

We did not include the lumbar infusion test, a predictive test with similar performance as the CSF TT [17, 52], in our standard protocol where it is reserved for patients in which we expect difficulties assessing symptom improvement at the CSF TT such as patients who cannot undergo the standard CSF TT protocol. It is reasonable to believe that our protocol for selecting patients for shunt surgery has affected outcomes compared to if we would have used other predictive tests alone or in combination. However, our selection procedure adopts to other studies and guidelines [10, 17]. Our test protocol comprises a battery of gait and balance tests for a more sensitive assessment approach and offering an understanding of mobility changes in iNPH from several aspects, providing deeper insight into selecting surgery candidates. Included tests represent established physiotherapy tests for neurological disease with standardized procedures available in health care institutions.

Regarding sex differences, the present study indicated worse baseline performance among females compared to males which agrees with previous studies [19, 53, 54], and the cardiovascular disease prevalence was higher in men, in line with Norwegian registry data [55]. However, the magnitude of improvement as measured by these tests post-CSF TT was not significantly different between sexes, mirroring previous surgical outcome findings [19]. Our study therefore suggests that the tests included here are sensitive for capturing changes in iNPH patients of both sexes.

We aimed to explore CSF TT results in a selected group of patients diagnosed with iNPH and subjected to shunt surgery and included patients selected for shunt surgery based on tap test results, not exploring CSF TT outcomes in patients whose tap test was deemed negative, hence not operated on. Thus, this study cannot estimate the CSF TT outcome in an unselected population of people with suspected iNPH.

The overall response rate of 55% reported here is lower than in many earlier studies, including studies from our center [56]. Patients included here comprise a selected group of iNPH patients with more uncertain clinical and MRI features and, possibly, worse prognosis, despite a positive CSF TT which may illustrate that also other factors than outcome of CSF TT may influence postoperative outcomes.

Further, our definition of a positive CSF TT defines the study population. The10MWT is suggested as a measure for CSF TT outcome, however, no consensus exists on the use of a specific cutoff in improvement. In the most recent clinical guidelines for iNPH, the Japanese iNPH Guidelines [10], several tests are suggested and the value of also assessing changes in gait quality are stressed. Therefore, this holistic approach for assessing CSF TT response adheres to current clinical guidelines and, in our experience, represents a useful clinical tool in the workup of iNPH patients.

This present study reinforces the utility of expanding standard CSF TT assessment to include challenging static balance tasks – such as foam cushion standing or tandem stance, challenging walking direction using 3MBW, and evaluating functional capacity with 6MWT. These tests provided greater sensitivity to change than standard measures like 10MWT and TUG. Including these tests may improve the identification of CSF TT responders and provide a more comprehensive evaluation of both gait and balance impairment to guide surgical decisions.

### Limitations & strengths

The present study has several limitations and strengths. First, while potential ceiling effects in static balance tests are recognized, we mitigated this issue through including a comprehensive battery of tests with varying difficulty levels, which enhanced our ability to detect changes. Another limitation is that the selected group was not compared to those who directly underwent surgery (without tap-test), limiting the scope of comparative analysis. Neither did the current study compare changes in outcome after the CSF-TT, depending on shunt responders vs. non-responders.

Despite these constraints, the study presents significant strengths. We conducted a comprehensive physiotherapy-based assessment protocol that captured a broad spectrum of gait and balance functions, including novel tests such as 3MBW test, offering detailed insights into mobility and postural control changes. Furthermore, by evaluating sex-based differences we provide support of a uniform strategy across diverse patient characteristics.

### Implications and future directions

The finding suggests that a comprehensive physiotherapy-based CSF TT protocol may have enabled the identification of patients who would otherwise have been excluded from shunt surgery based on the outcome in a single clinical test such as the 10MWT alone.

## Conclusions

The present study demonstrates the utility of a comprehensive physiotherapy-based assessment protocol for detecting changes following CSF TT in patients with suspected iNPH. The 3MBW was the most sensitive measure, while the 6MWT provided important complementary insights alongside the commonly used 10-meter walk test. Static balance assessment along with Romberg’s test, foam cushion stances (heels and feet together) and tandem stance demonstrated significant improvement following CSF TT supporting the notion that also balance tests could be included in the CSF TT protocol. No sex-based differences were observed after adjusting for baseline performance. These findings may support a more refined approach to CSF TT interpretation and patient selection for shunt surgery, underscoring the importance of incorporating a broader range of physiotherapy measures into clinical assessments.

## Supplementary Information

Below is the link to the electronic supplementary material.

Supplementary Material 1

## Data Availability

According to Swedish regulations, the dataset generated within this study’s framework cannot be made publicly available for ethical and legal reasons. The research data can be made available on reasonable request to the corresponding author.

## Abbreviations

iNPH: Idiopathic Normal Pressure Hydrocephalus
CSF TT: Cerebrospinal fluid tap test
MRI: Magnetic Resonance Imaging
CT: Computed tomography
LP: Lumbar puncture
10MWT: 10-Meter walk test
TUG: Timed-up and go
EO/EC: Eyes open/eyes closed
3MBW: 3-Meter backward walk
6MWT: 6-Meter walking test
30sCST: 30-second chair stand test
MMSE: Mini-Mental State Examination
SGPALS: Saltin and Grimby Physical Activity Level Scale
mRS: modified Rankin Scale
BMI: Body Mass Index

## References

[1] Constantinescu C, Wikkelsø C, Westman E, et al. Prevalence of possible idiopathic normal pressure hydrocephalus in Sweden: a population-based MRI study in 791 70-year-old participants. Neurology. 2024;102(2). 10.1212/WNL.0000000000208037. 10.1212/WNL.0000000000208037 PMC10962905 38165321

[2] Relkin N, Marmarou A, Klinge P, Bergsneider M, McL Black P. Diagnosing idiopathic normal-pressure hydrocephalus. Neurosurgery. 2005;57:S24–216. 10.1227/01.NEU.0000168185.29659.C5. 16160425 10.1227/01.neu.0000168185.29659.c5

[3] Möhwald K, Wuehr M, Decker J, et al. Quantification of pathological gait parameter thresholds of idiopathic normal pressure hydrocephalus patients in clinical gait analysis. Sci Rep. 2022;12(1). 10.1038/s41598-022-22692-1. 10.1038/s41598-022-22692-1 PMC9622747 36316420

[4] Agerskov S, Hellström P, Andrén K, Kollén L, Wikkelsö C, Tullberg M. The phenotype of idiopathic normal pressure hydrocephalus-a single center study of 429 patients. J Neurol Sci. 2018;391:54–60. 10.1016/j.jns.2018.05.022. 30103972 10.1016/j.jns.2018.05.022

[5] Rydja J, Pohl P, Eleftheriou A, Lundin F. Gait characteristics in idiopathic normal pressure hydrocephalus: a controlled study using an inertial sensor system. PLoS ONE. 2025;20(2 February). 10.1371/journal.pone.0317901. 10.1371/journal.pone.0317901 PMC11864528 40009597

[6] Blomsterwall E, Bilting M, Stephensen H, Wikkelsö C. Gait abnormality is not the only motor disturbance in normal pressure hydrocephalus. Scand J Rehabil Med. 1995;27(4):205–9. 8650504

[7] Nikaido Y, Urakami H, Akisue T, et al. Associations among falls, gait variability, and balance function in idiopathic normal pressure hydrocephalus. Clin Neurol Neurosurg. 2019;183. 10.1016/j.clineuro.2019.105385. 10.1016/j.clineuro.2019.105385 31207457

[8] Gallia GL, Rigamonti D, Williams MA. The diagnosis and treatment of idiopathic normal pressure hydrocephalus. Nat Clin Pract Neurol. 2006;2(7):375–81. 10.1038/ncpneuro0237. 16932588 10.1038/ncpneuro0237

[9] Wikkelsö C, Andersson H, Blomstrand C, Lindqvist G, Svendsen P. Predictive value of the cerebrospinal fluid tap-test. Acta Neurol Scand. 1986;73(6):566–73. 10.1111/j.1600-0404.1986.tb04601.x. 3751498 10.1111/j.1600-0404.1986.tb04601.x

[10] Nakajima M, Yamada S, Miyajima M, et al. Guidelines for management of idiopathic normal pressure hydrocephalus (Third edition): endorsed by the Japanese society of normal pressure hydrocephalus. Neurol Med Chir (Tokyo). 2021;61(2):63–97. 10.2176/nmc.st.2020-0292. 33455998 10.2176/nmc.st.2020-0292 PMC7905302

[11] Marmarou A, Bergsneider M, Klinge P, Relkin N, Black PML. INPH guidelines, part III: the value of supplemental prognostic tests for the preoperative assessment of idiopathic normal-pressure hydrocephalus. Neurosurgery. 2005;57(3 SUPPL). 10.1227/01.NEU.0000168184.01002.60. 10.1227/01.neu.0000168184.01002.60 16160426

[12] Oztop Cakmak O. Comparative assessment of gait and balance in patients with Parkinson’s disease and normal pressure Hydrocephalus. SiSli Etfal Hastanesi Tip Bulteni / The Medical Bulletin of Sisli Hospital. Published online 2023. 10.14744/semb.2023.79990. 10.14744/SEMB.2023.79990 PMC10600622 37899810

[13] Dumarey NE, Massager N, Laureys S, Goldman S. Voxel-based assessment of spinal tap test-induced regional cerebral blood flow changes in normal pressure Hydrocephalus. http://journals.lww.com/nuclearmedicinecomm. 10.1097/01.mnm.0000170937.90958.22 16096578

[14] Allali G, Laidet M, Armand S, Assal F. Brain comorbidities in normal pressure hydrocephalus. Eur J Neurol. 2018;25(3):542–8. 10.1111/ene.13543. 29222955 10.1111/ene.13543 PMC5947755

[15] Köster H, Müller-Schmitz K, Kolman AGJ, Seitz RJ. Deficient visuomotor hand coordination in normal pressure hydrocephalus. J Neurol. 2021;268(8):2843–50. 10.1007/s00415-021-10445-5. 33594453 10.1007/s00415-021-10445-5 PMC8289764

[16] Davis A, Gulyani S, Manthripragada L, Luciano M, Moghekar A, Yasar S. Evaluation of the effect comorbid Parkinson syndrome on normal pressure hydrocephalus assessment. Clin Neurol Neurosurg. 2021;207. 10.1016/j.clineuro.2021.106810. 10.1016/j.clineuro.2021.106810 34280677

[17] Wikkelsø C, Hellström P, Klinge PM, Tans JTJ. The European iNPH multicentre study on the predictive values of resistance to CSF outflow and the CSF tap test in patients with idiopathic normal pressure hydrocephalus. J Neurol Neurosurg Psychiatry. 2013;84(5):562–8. 10.1136/jnnp-2012-303314. 23250963 10.1136/jnnp-2012-303314

[18] Hereitová I, Griffa A, Allali G, Dorňák T. Gait characteristics in idiopathic normal pressure hydrocephalus: a review on the effects of CSF tap test and shunt surgery. Eur J Med Res. 2024;29(1):633. 10.1186/s40001-024-02162-2. 39734225 10.1186/s40001-024-02162-2 PMC11684308

[19] Sundström N, Rydja J, Virhammar J, Kollén L, Lundin F, Tullberg M. The timed up and go test in idiopathic normal pressure hydrocephalus: a nationwide study of 1300 patients. Fluids Barriers CNS. 2022;19(1). 10.1186/s12987-021-00298-5. 10.1186/s12987-021-00298-5 PMC8750754 35012586

[20] El Ahmadieh TY, Wu EM, Kafka B, et al. Lumbar drain trial outcomes of normal pressure hydrocephalus: A single-center experience of 254 patients. J Neurosurg. 2020;132(1):306–12. 10.3171/2018.8.JNS181059. 30611143 10.3171/2018.8.JNS181059

[21] Ravdin LD, Katzen HL, Jackson AE, Tsakanikas D, Assuras S, Relkin NR. Features of gait most responsive to tap test in normal pressure hydrocephalus. Clin Neurol Neurosurg. 2008;110(5):455–61. 10.1016/j.clineuro.2008.02.003. 18359152 10.1016/j.clineuro.2008.02.003 PMC2690636

[22] Stolze H, Kuhtz-Buschbeck JP, Ècke HD, et al. Gait analysis in idiopathic normal pressure hydrocephalus ± which parameters respond to the CSF tap test? https://www.elsevier.com/locate/clinph 10.1016/s1388-2457(00)00362-x 10964082

[23] Ishikawa M, Hashimoto M, Mori E, Kuwana N, Kazui H. The value of the cerebrospinal fluid tap test for predicting shunt effectiveness in idiopathic normal pressure hydrocephalus. Fluids Barriers CNS. 2012;9(1). 10.1186/2045-8118-9-1. 10.1186/2045-8118-9-1 PMC3293050 22239832

[24] Bovonsunthonchai S, Witthiwej T, Vachalathiti R, et al. Clinical improvements in temporospatial gait variables after a spinal tap test in individuals with idiopathic normal pressure hydrocephalus. Sci Rep. 2024;14(1). 10.1038/s41598-024-52516-3. 10.1038/s41598-024-52516-3 PMC10808249 38267518

[25] Schniepp R, Trabold R, Romagna A, et al. Walking assessment after lumbar puncture in normal-pressure hydrocephalus: A delayed improvement over 3 days. J Neurosurg. 2017;126(1):148–57. 10.3171/2015.12.JNS151663. 26991388 10.3171/2015.12.JNS151663

[26] Klinge P, Hellström P, Tans J, Wikkelsø C. One-year outcome in the European multicentre study on iNPH. Acta Neurol Scand. 2012;126(3):145–53. 10.1111/j.1600-0404.2012.01676.x. 22571428 10.1111/j.1600-0404.2012.01676.x

[27] Lanska DJ, Goetz CG. Historical neurology Romberg’s sign development, adoption, and adaptation in the 19th Century. 2000. https://www.neurology.org. 10.1212/wnl.55.8.1201 11071500

[28] Nemanich ST, Duncan RP, Dibble LE, et al. Predictors of gait speeds and the relationship of gait speeds to falls in men and women with Parkinson disease. Parkinsons Dis Published Online. 2013. 10.1155/2013/141720. 10.1155/2013/141720 PMC3687488 23841020

[29] Mbada CE, Afolabi AD, Akinkuoye A, et al. Reference values for 3-Meter backward walk test among apparently healthy adults. Med Principles Pract. 2023;32(6):351–7. 10.1159/000534649. 10.1159/000534649 PMC10727517 37852188

[30] Guyatt GH, Sullivan MJ, Thompson PJ, Fallen EL, Pugsley SO, Taylor DW, Berman LB. The 6-minute walk: a new measure of exercise capacity in patients with chronic heart failure. Can Med Assoc J. 1985 Apr 15;132(8):919-23. PMC1345899 3978515

[31] Podsiadlo JD, Bscpt S, Richardson MDJ. The timed up & Go: a test of basic functional mobilitv for Frail Elderlv Persons. 1991;39. 10.1111/j.1532-5415.1991.tb01616.x 1991946

[32] Jones CJ, Rikli RE, Beam WC. A 30-s chair-stand test as a measure of lower body strength in community-residing older adults. Res Q Exerc Sport. 1999;70(2):113–9. 10.1080/02701367.1999.10608028. 10380242 10.1080/02701367.1999.10608028

[33] Hellström P, Klinge P, Tans J, Wikkelsø C. A new scale for assessment of severity and outcome in iNPH. Acta Neurol Scand. 2012;126(4):229–37. 10.1111/j.1600-0404.2012.01677.x. 22587624 10.1111/j.1600-0404.2012.01677.x

[34] Crum RM, Anthony JC, Bassett SS, Folstein MF. Population-based norms for the mini-mental state examination by age and educational level. http://jama.jamanetwork.com/. 8479064

[35] Van Swieten JC, Koudstaal PJ, Visser MC, Schouten HJA, Van Gijn J. Interobserver agreement for the assessment of handicap in stroke patients. http://ahajournals.org. 10.1161/01.str.19.5.604 3363593

[36] Grimby G, Börjesson M, Jonsdottir IH, Schnohr P, Thelle DS, Saltin B. The Saltin-Grimby physical activity level scale and its application to health research. Scand J Med Sci Sports. 2015;25:119–25. 10.1111/sms.12611. 26589125 10.1111/sms.12611

[37] Ropper AH. Refined Romberg test. Can J Neurol Sci / J Canadien Des Sci Neurologiques. 1985;12(3):282. 10.1017/S0317167100047193. 10.1017/s0317167100047193 2996736

[38] Rydja J, Eleftheriou A, Lundin F. Evaluating the cerebrospinal fluid tap test with the Hellström iNPH scale for patients with idiopathic normal pressure hydrocephalus. Fluids Barriers CNS. 2021;18(1). 10.1186/s12987-021-00252-5. 10.1186/s12987-021-00252-5 PMC8025497 33827613

[39] Pearce RKB, Gontsarova A, Richardson D, et al. Shunting for idiopathic normal pressure hydrocephalus. Cochrane Database Syst Reviews. 2024;2024(8). 10.1002/14651858.CD014923.pub2. 10.1002/14651858.CD014923.pub2 PMC11301990 39105473

[40] Luciano M, Holubkov R, Williams MA, et al. Placebo-Controlled effectiveness of idiopathic normal pressure hydrocephalus shunting: A randomized pilot trial. Neurosurgery. 2023;92(3):481–9. 10.1227/neu.0000000000002225. 36700738 10.1227/neu.0000000000002225 PMC9904195

[41] Matsuoka T, Fujimoto K, Kawahara M. Comparison of comfortable and maximum walking speed in the 10-meter walk test during the cerebrospinal fluid tap test in iNPH patients: a retrospective study. Clin Neurol Neurosurg. 2022;212. 10.1016/j.clineuro.2021.107049. 10.1016/j.clineuro.2021.107049 34871990

[42] Carter V, Jain T, James J, Cornwall M, Aldrich A, De Heer HD. The 3-m backwards walk and retrospective falls: diagnostic accuracy of a novel clinical measure. J Geriatr Phys Ther. 2019;42(4):249–55. 10.1519/JPT.0000000000000149. 29095771 10.1519/JPT.0000000000000149

[43] Fritz NE, Worstell AM, Kloos AD, Siles AB, White SE, Kegelmeyer DA. Backward walking measures are sensitive to age-related changes in mobility and balance. Gait Posture. 2013;37(4):593–7. 10.1016/j.gaitpost.2012.09.022. 23122938 10.1016/j.gaitpost.2012.09.022

[44] È WC, Blomsterwall E, Svantesson U, et al. Postural disturbance in patients with normal pressure hydrocephalus. 2000;102. 10.1034/j.1600-0404.2000.102005284.x 11083504

[45] Bäcklund T, Frankel J, Israelsson H, Malm J, Sundström N. Trunk sway in idiopathic normal pressure hydrocephalus—Quantitative assessment in clinical practice. Gait Posture. 2017;54:62–70. 10.1016/j.gaitpost.2017.02.017. 28259041 10.1016/j.gaitpost.2017.02.017

[46] Taulbee L, Yada T, Graham L, et al. Use of backward walking speed to screen dynamic balance and mobility deficits in older adults living independently in the community. J Geriatr Phys Ther. 2021;44(4):189–97. 10.1519/JPT.0000000000000290. 33534335 10.1519/JPT.0000000000000290

[47] Rose DK, Demark L, Fox EJ, Clark DJ, Wludyka P. A backward walking training program to improve balance and mobility in acute stroke: A pilot randomized controlled trial. J Neurol Phys Ther. 2018;42(1):12–21. 10.1097/NPT.0000000000000210. 29232308 10.1097/NPT.0000000000000210

[48] Soke F, Aydin F, Karakoc S, et al. Effects of backward walking training on balance, gait, and functional mobility in people with multiple sclerosis: A randomized controlled study. Mult Scler Relat Disord. 2023;79. 10.1016/j.msard.2023.104961. 10.1016/j.msard.2023.104961 37683559

[49] Rydja J, Kollén L, Ulander M, Tullberg M, Lundin F. Physical capacity and activity in patients with idiopathic normal pressure hydrocephalus. Front Neurol. 2022;13. 10.3389/fneur.2022.845976. 10.3389/fneur.2022.845976 PMC8996117 35418936

[50] Nikaido Y, Kajimoto Y, Tucker A, et al. Intermittent gait disturbance in idiopathic normal pressure hydrocephalus. Acta Neurol Scand. 2018;137(2):238–44. 10.1111/ane.12853. 29023635 10.1111/ane.12853

[51] Virhammar J, Cesarini KG, Laurell K. The CSF tap test in normal pressure hydrocephalus: evaluation time, reliability and the influence of pain. Eur J Neurol. 2012;19(2):271–6. 10.1111/j.1468-1331.2011.03486.x. 21801282 10.1111/j.1468-1331.2011.03486.x

[52] Raneri F, Zella MAS, Di Cristofori A, Zarino B, Pluderi M, Spagnoli D. Supplementary tests in idiopathic normal pressure hydrocephalus: A Single-Center experience with a combined lumbar infusion test and tap test. World Neurosurg. 2017;100:567–74. 10.1016/j.wneu.2017.01.003. 28089835 10.1016/j.wneu.2017.01.003

[53] Andersson J, Rosell M, Kockum K, Lilja-Lund O, Söderström L, Laurell K. Prevalence of idiopathic normal pressure hydrocephalus: A prospective, population based study. PLoS One Public Libr Science. 2019;14(5). 10.1371/journal.pone.0217705. 10.1371/journal.pone.0217705 PMC6541279 31141553

[54] Jaraj D, Rabiei K, Marlow T, Jensen C, Skoog I, Wikkelsø C. Prevalence of idiopathic normal-pressure hydrocephalus. 2014. https://www.neurology.org 10.1212/WNL.0000000000000342 PMC4001197 24682964

[55] Eide PK, Pripp AH. Increased prevalence of cardiovascular disease in idiopathic normal pressure hydrocephalus patients compared to a population-based cohort from the HUNT3 survey. Fluids Barriers CNS. 2014;11(1). 10.1186/2045-8118-11-19. 10.1186/2045-8118-11-19 PMC4150119 25180074

[56] Andrén K, Wikkelsø C, Laurell K, Kollén L, Hellström P, Tullberg M. Symptoms and signs did not predict outcome after surgery: a prospective study of 143 patients with idiopathic normal pressure hydrocephalus. J Neurol. 2024;271(6):3215–26. 10.1007/s00415-024-12248-w. 38438818 10.1007/s00415-024-12248-w PMC11136756

