# Supplementary material for: Sensitivity of physiotherapy-based clinical tests in detecting change in gait and balance performance following a 50 mL CSF tap test in idiopathic normal pressure hydrocephalus
Source: Fluids Barriers CNS. 2026 Feb 17;23:35. doi: 10.1186/s12987-026-00776-8 (PMC12931076; doi:10.1186/s12987-026-00776-8)
Supplement: Supplementary file 1 — Supplementary Material 1 [file 12987_2026_776_MOESM1_ESM.docx]

**Sensitivity of Physiotherapy-Based Clinical Tests in Detecting Gait and Balance Performance Following a 50 mL CSF Tap Test in Idiopathic Normal Pressure Hydrocephalus**

Following CSF TT, change within female and male groups, and between sexes shown in Supplementary Tables 1 and 2.

Supplementary. Table 1: Static Balance and 30-Second Chair Stand Test Change After CSF TT in Female and Male Groups.

|  | Sex | Number of missing | Pre-tap | Post-tap | Within group | | Between groups | | | |
| --- | --- | --- | --- | --- | --- | --- | --- | --- | --- | --- |
|  |  |  |  |  | Mean difference (95% CI) | p-value | Adjusted mean difference* (95% CI) of change | p-value  of change | p-value  Pre-CSF TT | p-value  Post-CSF TT |
| Heels together, sec | Female | 1 | 25.6 (9.3) | 27.1 (8.4) | 1.5 (-0.4 - 3.4) | ns | -1.62 (-3.99 - 0.75) | ns | ns | ns |
|  | Male | 2 | 25.5 (9.3) | 28.7 (5.1) | 3.2 (0.7 - 5.6) | **0.012** |  |  |  |  |
| Heels together-EC, sec | Female | 3 | 19 (12.4) | 23.1 (10.9) | 3.9 (1.2 - 6.5) | **0.005** | 0.09 (-3.19 - 3.37) | ns | ns | ns |
|  | Male | 2 | 19.1 (12.3) | 22.9 (10.8) | 3.8 (0.9 - 6.6) | **0.01** |  |  |  |  |
| Feet together, sec | Female | 1 | 24.2 (10.8) | 25.8 (9.5) | 1.7 (-0.8 - 4.1) | ns | -0.39 (-3.15 - 2.38) | ns | ns | ns |
|  | Male | 2 | 23.8 (10.5) | 26 (9) | 2.2 (0 - 4.4) | **0.047** |  |  |  |  |
| Romberg, sec | Female | 3 | 15.6 (13.6) | 20.4 (12.1) | 4.6 (1.7 - 7.4) | **0.002** | 0.31 (-3.07 - 3.68) | ns | ns | ns |
|  | Male | 3 | 12.3 (12.4) | 17.7 (12.4) | 5.2 (2.7 - 7.6) | **<.001** |  |  |  |  |
| Heels together-foam cushion, sec | Female | 5 | 14.3 (13) | 21.2 (12.8) | 6.5 (2.5 - 10.4) | **0.002** | 3.31 (-0.69 - 7.30) | ns | ns | ns |
|  | Male | 6 | 19.3 (13.6) | 21.6 (12.6) | 1.9 (0.1 - 3.7) | **0.035** |  |  |  |  |
| Heels together-foam cushion-EC, sec | Female | 10 | 3. (7.2) | 7.4 (10.1) | 4.1 (1.1 - 7.1) | **0.009** | 1.92 (-1.45 - 5.29) | ns | **0.032** | ns |
|  | Male | 7 | 7.3 (10.9) | 9.3 (11.5) | 1.8 (0.2 - 3.3) | **0.029** |  |  |  |  |
| Feet together- foam cushion, sec | Female | 6 | 12.9 (13.1) | 18.3 (13.8) | 4.9 (1.4 - 8.5) | **0.008** | 1.13 (-3.03 - 5.29) | ns | ns | ns |
|  | Male | 7 | 15.6 (13.8) | 19.5 (13.5) | 3.1 (0.6 - 5.7) | **0.018** |  |  |  |  |
| Romberg-foam cushion, sec | Female | 10 | 1.6 (5.2) | 4.6 (8.5) | 3.0 (0.5 - 5.4) | **0.02** | 0.35 (-2.65 - 3.36) | ns | ns | ns |
|  | Male | 7 | 3.7 (7.5) | 6.8 (10.4) | 2.8 (1.1 - 4.6) | **0.002** |  |  |  |  |
| Tandem stance-EO, sec | Female | 9 | 4.7 (7.2) | 7.4 (10.8) | 3.0 (0.6 - 5.4) | **0.016** | -0.11 (-3.16 - 2.94) | ns | ns | ns |
|  | Male | 6 | 5.5 (8.1) | 8.9 (10.5) | 3.2 (1.2 - 5.1) | **0.002** |  |  |  |  |
| Tandem stance-EC, sec | Female | 9 | 0.6 (2.1) | 0.5 (1.4) | -0.0 (-0.6 - 0.5) | ns | -0.52 (-1.20 - 0.17) | ns | ns | ns |
|  | Male | 7 | 0.4 (1.5) | 1.0 (2.5) | 0.5 (-0.0 - 1.1) | ns |  |  |  |  |
| Right leg stance-EO, sec | Female | 9 | 1.1 (2.6) | 1.9 (2.5) | 0.7 (-0.7 - 2.1) | ns | 0.36 (-1.21 - 1.92) | ns | ns | ns |
|  | Male | 7 | 2.2 (6.1) | 2.6 (5.6) | 0.3 (-0.4 - 0.9) | ns |  |  |  |  |
| Left leg stance-EO, sec | Female | 8 | 1.2 (2.7) | 1.9 (3.6) | 0.7 (-0.1 - 1.5) | ns | 0.57 (-0.89 - 2.03) | ns | ns | ns |
|  | Male | 7 | 1.3 (4.5) | 1.5 (3.9) | 0.1 (-1.3 - 1.5) | ns |  |  |  |  |
| 30sCST, number of stands | Female | 6 | 3.4 (4.1) | 4.6 (4.1) | 0.9 (0.4 - 1.4) | **<.001** | -0.30 (-0.94 - 0.34) | ns | ns | ns |
|  | Male | 4 | 4.6 (4.1) | 5.6 (4.4) | 1.2 (0.8 - 1.6) | **<.001** |  |  |  |  |
| Descriptive data are presented as mean (SD)  * Adjusted for pre-test values.  Abbreviations: EO, eyes open; EC, eyes closed; 30sCST, 30-second chair stand test; CI, confidence interval. | | | | | | | | | | |

Supplementary Table 2: Walking Tests Change After CSF TT in Female and Male Groups.

|  | Sex | Number of missing | Pre-tap | Post-tap | Fold change (95% CI) | p-value | Improved, n (%) | p-value | Between sexes | | | |
| --- | --- | --- | --- | --- | --- | --- | --- | --- | --- | --- | --- | --- |
|  |  |  |  |  |  |  |  |  | Adjusted fold change* (95% CI) | p-value of change | p-value  Pre-CSF TT | p-value  Post CSF-TT |
| 10MWT-SS, sec | Female | 3 | 18.0 (0.55) | 13.7 (0.39) | 0.76 (0.67 - 0.85) | **<.001** | 32 (84%) | **<.001** | 0.98 (0.88 - 1.08) | ns | ns | ns |
|  | Male | 2 | 17.0 (0.52) | 13.5 (0.39) | 0.79 (0.74 - 0.85) | **<.001** | 47 (90%) | **<.001** |  |  |  |  |
| 10MWT-SS, sec, best | Female | 3 | 17.3 (0.52) | 13.4 (0.39) | 0.77 (0.69 - 0.87) | **<.001** | 32 (84%) | **<.001** | 0.99 (0.89 - 1.09) | ns | ns | ns |
|  | Male | 2 | 16.6 (0.51) | 13.2 (0.39) | 0.80 (0.74 - 0.85) | **<.001** | 45 (87%) | **<.001** |  |  |  |  |
| 10MWT-SS, steps | Female | 3 | 25.7 (0.34) | 21.9 (0.25) | 0.85 (0.79 - 0.91) | **<.001** | 29 (76%) | **0.002** | 1.02 (0.95 - 1.09 | ns | ns | ns |
|  | Male | 2 | 25.3 (0.42) | 21.3 (0.29) | 0.84 (0.79 - 0.90) | **<.001** | 41 (79%) | **<.001** |  |  |  |  |
| 10MWT-SS, steps, best | Female | 3 | 25.1 (0.33) | 21.6 (0.25) | 0.86 (0.80 - 0.92) | **<.001** | 29 (76%) | **0.002** | 1.02 (0.95 - 1.09) | ns | ns | ns |
|  | Male | 2 | 24.8 (0.42) | 21.0 (0.29) | 0.85 (0.79 - 0.90) | **<.001** | 40 (77%) | **<.001** |  |  |  |  |
| 10MWT-MS, sec | Female | 11 | 11.6 (0.41) | 9.8 (0.32) | 0.84 (0.78 - 0.91) | **<.001** | 27 (90%) | **<.001** | 1.01 (0.93 - 1.09) | ns | ns | ns |
|  | Male | 12 | 10.7 (0.39) | 9.2 (0.31) | 0.86 (0.81- 0.91) | **<.001** | 29 (69%) | **0.020** |  |  |  |  |
| 10MWT-MS, steps | Female | 11 | 20.8 (0.25) | 18.8 (0.24) | 0.91 (0.86 - 0.96) | **<.001** | 24 (80%) | **0.001** | 1.04 (0.93 - 1.17) | ns | ns | ns |
|  | Male | 11 | 19.1 (0.26) | 16.9 (0.22) | 0.89 (0.85 - 0.92) | **<.001** | 34 (79%) | **<.001** |  |  |  |  |
| 3MBW, sec | Female | 3 | 18.4 (0.75) | 11.5 (0.66) | 0.63 (0.55 - 0.71) | **<.001** | 35 (92%) | **<.001** | 0.93 (0.81 - 1.06) | ns | ns | ns |
|  | Male | 4 | 14.1 (0.75) | 10.2 (0.60) | 0.73 (0.66 - 0.80) | **<.001** | 41 (82%) | **<.001** |  |  |  |  |
| 3MBW, steps | Female | 3 | 23.2 (0.61) | 16.9 (0.56) | 0.73 (0.65 - 0.81) | **<.001** | 36 (95%) | **<.001** | 0.95 (0.84 - 1.07) | ns | ns | ns |
|  | Male | 4 | 19.1 (0.59) | 15.3 (0.52) | 0.80 (0.74 - 0.86) | **<.001** | 39 (78%) | **<.001** |  |  |  |  |
| TUG, sec | Female | 1 | 28.6 (0.81) | 20.8 (0.67) | 0.73 (0.65 - 0.82) | **<.001** | 36 (90%) | **<.001** | 1.04 (0.93 - 1.17) | ns | ns | ns |
|  | Male | 2 | 23.9 (0.69) | 17.6 (0.49) | 0.74 (0.68 - 0.80) | **<.001** | 50 (96%) | **<.001** |  |  |  |  |
| TUG, steps | Female | 2 | 26.1 (0.48) | 21.3 (0.35) | 0.82 (0.74 - 0.90) | **<.001** | 31 (79%) | **<.001** | 1.02 (0.93 - 1.11) | ns | ns | ns |
|  | Male | 2 | 25.6 (0.50) | 20.8 (0.36) | 0.81 (0.76 - 0.87) | **<.001** | 42 (81%) | **<.001** |  |  |  |  |
| 6 MWT, meters | Female | 8 | 194.0 (0.66) | 236.5 (0.61) | 1.22 (1.11 - 1.34) | **<.001** | 29 (88%) | **<.001** | 0.98 (0.88 - 1.08) | ns | ns | ns |
|  | Male | 2 | 169.8 (0.91) | 214.7 (0.87) | 1.26 (1.20 - 1.34) | **<.001** | 47 (90%) | **<.001** |  |  |  |  |
| Descriptive data are presented as geometric mean (coefficient of variation)  All outcomes were log-transformed prior to analysis and results presented as fold change  Abbreviations: CI, confidence interval; FC, fold change. | | | | | | | | | | | | |

Supplementary Table 3: Number of missing data in descriptive data

|  | | Total | Female | Male |
| --- | --- | --- | --- | --- |
| Age, years | | 0 | 0 | 0 |
| Sex, male (M%) | | 0 | 0 | 0 |
| BMI, kg/m^2^ | | 4 | 2 | 2 |
| Disease duration, months | | 4 | 2 | 2 |
| Comorbidities/disease, n (%) | Hypertension | 0 | 0 | 0 |
|  | Diabetes | 0 | 0 | 0 |
|  | Cardiovascular | 0 | 0 | 0 |
|  | Hyperlipidemia | 20 | 10 | 10 |
|  | Ischemic heart | 19 | 10 | 9 |
|  | Other hearth | *16* | 10 | 6 |
|  | Cerebrovascular | *15* | 9 | 6 |
|  | Any vascular risk | *19* | 10 | 9 |
|  | Other neurological | *33* | 14 | 19 |
| Falls, number within 3 months | | *17* | 5 | 12 |
| Shunt surgery type, n (%) | VP | *0* | 0 | 0 |
|  | VA | 0 | 0 | 0 |
| iNPH scale, score | Total | 0 | 0 | 0 |
|  | Gait domain | 1 | 1 | 0 |
|  | Neuropsychiatric domain | *0* | 0 | 0 |
|  | Balance domain | 0 | 0 | 0 |
|  | Continence domain | 0 | 0 | 0 |
| MMSE score | | 0 | 0 | 0 |
| mRS | | 0 | 0 | 0 |
| SGPALS | | 26 | 11 | 15 |
